# Synthetic redesign of *Escherichia coli* W for faster metabolism of sugarcane molasses

**DOI:** 10.1186/s12934-024-02520-z

**Published:** 2024-09-09

**Authors:** Gi Yeon Kim, Jina Yang, Yong Hee Han, Sang Woo Seo

**Affiliations:** 1 Interdisciplinary Program in Bioengineering, Seoul National University, 1 Gwanak-ro, Gwanak-gu, Seoul, 08826 South Korea; 2 Department of Chemical Engineering, Jeju National University, 102, Jejudaehak-ro, Jeju-si, Jeju-do 63243 Korea; 3 School of Biological Sciences and Biotechnology, Graduate School, and School of Biological Sciences and Technology, Chonnam National University, Yongbong-ro 77, Gwangju, 61186 South Korea; 4 School of Chemical and Biological Engineering, Seoul National University, 1 Gwanak-ro, Gwanak-gu, Seoul, 08826 South Korea; 5 Institute of Chemical Processes, and Bio-MAX Institute, and Institute of Bio Engineering, Seoul National University, 1 Gwanak-ro, Gwanak-gu, Seoul, 08826 South Korea

**Keywords:** Sugarcane molasses, Sugar uptake, Carbon catabolite repression, 3-hydroxypropionic acid

## Abstract

**Background:**

Sugarcane molasses, rich in sucrose, glucose, and fructose, offers a promising carbon source for industrial fermentation due to its abundance and low cost. However, challenges arise from the simultaneous utilization of multiple sugars and carbon catabolite repression (CCR). Despite its nutritional content, sucrose metabolism in *Escherichia coli*, except for W strain, remains poorly understood, hindering its use in microbial fermentation. In this study, *E. coli* W was engineered to enhance sugar consumption rates and overcome CCR. This was achieved through the integration of a synthetically designed *csc* operon and the optimization of glucose and fructose co-utilization pathways. These advancements facilitate efficient utilization of sugarcane molasses for the production of 3-hydroxypropionic acid (3-HP), contributing to sustainable biochemical production processes.

**Results:**

In this study, we addressed challenges associated with sugar metabolism in *E. coli* W, focusing on enhancing sucrose consumption and improving glucose-fructose co-utilization. Through targeted engineering of the sucrose utilization system, we achieved accelerated sucrose consumption rates by modulating the expression of the *csc* operon components, *cscB*, *cscK*, *cscA*, and *cscR*. Our findings revealed that monocistronic expression of the *csc* genes with the deletion of *cscR*, led to optimal sucrose utilization without significant growth burden. Furthermore, we successfully alleviated fructose catabolite repression by modulating the binding dynamics of FruR with the fructose PTS regulon, enabling near-equivalent co-utilization of glucose and fructose. To validate the industrial applicability of our engineered strain, we pursued 3-HP production from sugarcane molasses. By integrating heterologous genes and optimizing metabolic pathways, we achieved improvements in 3-HP titers compared to previous studies. Additionally, glyceraldehyde-3-phosphate dehydrogenase (*gapA*) repression aids in carbon flux redistribution, enhancing molasses conversion to 3-HP.

**Conclusions:**

Despite limitations in sucrose metabolism, the redesigned *E. coli* W strain, adept at utilizing sugarcane molasses, is a valuable asset for industrial fermentation. Its synthetic *csc* operon enhances sucrose consumption, while mitigating CCR improves glucose-fructose co-utilization. These enhancements, coupled with repression of *gapA*, aim to efficiently convert sugarcane molasses into 3-HP, addressing limitations in sucrose and fructose metabolism for industrial applications.

**Supplementary Information:**

The online version contains supplementary material available at 10.1186/s12934-024-02520-z.

## Background

Sugarcane molasses is a byproduct of the sucrose production from sugarcane, and its utilization has many advantages, including its abundance, low cost, and high sugar content [1, 2]. The world’s annual yield of molasses reaches 55 million tons, with the global cane molasses market size setting to be about $8.1 billion by 2030 [2]. It contains not only sucrose but also glucose, fructose, and other nutrients such as vitamins, minerals, and amino acids, which leads to a potentially attractive carbon source for industrial fermentation processes. The utilization of renewable carbon feedstocks for bioprocesses is gaining significant attention, driven by environmental and economic considerations [3, 4]. Sucrose, a disaccharide composed of glucose and fructose, derived from sugarcane emerges as a promising renewable carbon source. While the precise composition varies among different sugarcane varieties, the proportion of sucrose within sugarcane molasses is significantly dominant among all carbon sources. Therefore, optimization of sucrose metabolism is crucial for effective utilization of sugarcane molasses.

Only a few non-pathogenic *E. coli* strains are known to utilize sucrose, including EC3132, B-62, and W [5–8]. *E. coli* W stands out as the sole well-known strain proficient in utilizing sucrose as a carbon source, displaying robust growth compared to alternative sources like glucose [9]. Given the limited ability of most *E. coli* strains to utilize sucrose, previous studies on sucrose metabolism primarily focused on engineering individual strains by introducing the sucrose uptake system from *E. coli* W [10–12]. Despite previous efforts, engineered strains for sucrose utilization often exhibited slow growth rates and phenotypic instability from unstable plasmid systems requiring antibiotics and cellular burden induced by high-copy number plasmids [6, 11, 13, 14]. Thus, there remains a critical need for efficient sucrose metabolism engineering in *E. coli*.

Sugarcane molasses contains not only sucrose, but glucose and fructose as carbon sources, posing challenges for simultaneous metabolism of multiple sugars. Carbon catabolite repression (CCR) is a regulatory mechanism where the presence of a preferred carbon source, often glucose, represses the utilization of alternative carbon sources, including fructose [15]. In *E. coli*, glucose is the preferred carbon substrate, and its abundance triggers CCR, leading to a reduction in the uptake of fructose. This regulatory hurdle limits the simultaneous utilization of multiple sugars, hindering the efficiency of certain bioprocesses [16]. Overcoming the CCR issue is crucial for engineering *E. coli* strains with improved capabilities for co-utilizing glucose and fructose, especially in industrial applications where the utilization of mixed sugars in common.

In this study, *E. coli* W was redesigned for faster consumption rates of sugars in molasses. Sugarcane molasses, a valuable resource rich in sucrose, glucose, and fructose, encounters a challenge in simultaneous utilization. The primary sucrose utilization system in *E. coli* W relies on non-PTS (phosphotransferase system), while glucose and fructose are predominantly utilized through PTS. A synthetically designed *csc* operon, which regulates the non-PTS sucrose metabolism, was shown to enhance the consumption rate of sucrose. Additionally, the co-utilization of glucose and fructose was improved by preventing the fructose repressor from binding to the fructose PTS regulon and controlling the expression level of the regulon. This approach solved the primary obstacle of CCR in glucose and fructose co-utilization. Furthermore, the increased consumption rates of sucrose, glucose, and fructose could help *E. coli* to efficiently utilize sugarcane molasses and convert it into 3-hydroxypropionic acid (3-HP). This approach aligns with the concept of utilizing renewable feedstocks, contributing to environmentally friendly and economically viable biochemical production processes.

## Methods

### Bacterial strains, plasmids, and reagents

*E. coli* strains and plasmids used in this study are listed in Table 1. Oligonucleotides used in this study are listed in Supplementary materials, Table S1. Reagents used for cell cultivation were purchased from BD Bioscience and Sigma Aldrich. Sugarcane molasses, containing a sucrose, glucose, and fructose ratio of approximately 4:1:1, was purchased from Plantation™ (Blackstrap Organic Unsulphured Molasses). Plasmid DNA was extracted using GeneAll^®^ Exprep™ Plasmid SV Kit, and PCR products were purified using Zymo Research DNA Clean & Concentrator or the GeneAll^®^ Expin Gel SV Kit. For gene cloning, Q5^®^ High-Fidelity DNA Polymerase, Quick Ligase, NEBuilder^®^ HiFi DNA Assembly Master Mix, and restriction enzymes were purchased from NEB (New England Biolabs).

**Table 1:** Strains and plasmids

Name	Relevant characteristics	Source
**Strains**		
Mach1-T1^R^	F − φ80(lacZ)ΔM15 ΔlacX74 hsdR(r − m +) ΔrecA1398 endA1 tonA	Invitrogen
*E. coli* W	Acid tolerant strain	KCTC1039
WΔ*cscR*	*E. coli* W/Δ*cscR*	This study
Pcsc_poly	*E. coli* W/Δ*cscBKAR*/pCDF-csc_poly	This study
Pcsc_mono	*E. coli* W/Δ*cscBKAR*/pCDF-csc_mono	This study
Rcsc	*E. coli* W/Δ*cscBKAR*::P_J23100_-synUTR_*cscB*_-*cscB* – P_J23106_-synUTR_*cscK*_*cscK* – P_*tac*_-synUTR_*cscA*_-*cscA*	This study
WΔ*fruR*	*E. coli* W/Δ*cscBKAR*::P_J23100_-synUTR_*cscB*_-*cscB* – P_J23106_-synUTR_*cscK*_-*cscK* – P_tac_-synUTR_*cscA*_-*cscA*/Δ*fruR*	This study
F1	*E. coli* W/Δ*cscBKAR*::P_J23100_-synUTR_*cscB*_-*cscB* – P_J23106_-synUTR_*cscK*_-*cscK* – P_*tac*_-synUTR_*cscA*_-*cscA*/P_Fru_-UTR_Fru_::P_*tac*_-synUTR_3,231_	This study
F2	*E. coli* W/Δ*cscBKAR*::P_J23100_-synUTR_*cscB*_-*cscB* – P_J23106_-synUTR_*cscK*_-*cscK* – P_*tac*_-synUTR_*cscA*_-*cscA*/P_Fru_-UTR_Fru_::P_*tac*_-synUTR_12,136_	This study
F3	*E. coli* W/Δ*cscBKAR*::P_J23100_-synUTR_*cscB*_-*cscB* – P_J23106_-synUTR_*cscK*_-*cscK* – P_*tac*_-synUTR_*cscA*_-*cscA*/P_Fru_-UTR_Fru_::P_*tac*_-synUTR_62,626_	This study
F4	*E. coli* W/Δ*cscBKAR*::P_J23100_-synUTR_*cscB*_-*cscB* – P_J23106_-synUTR_*cscK*_-*cscK* – P_tac_-synUTR_*cscA*_-*cscA*/P_Fru_-UTR_Fru_::P_*tac*_-synUTR_1,386,568_	This study
W_3HP	*E. coli* W/pUC-KGG/pACYC_B4	This study
F3_3HP	*E. coli* W/Δ*cscBKAR*::P_J23100_-synUTR_*cscB*_-*cscB* – P_J23106_-synUTR_*cscK*_-*cscK* – P_*tac*_-synUTR_*cscA*_-*cscA*/P_Fru_-UTR_Fru_::P_*tac*_-synUTR_62,626_/pUC-KGG/pACYC_B4	This study
G100_3HP	*E. coli* W/Δ*cscBKAR*::P_J23100_-synUTR_*cscB*_-*cscB* – P_J23106_-synUTR_*cscK*_*cscK* – P_*tac*_-synUTR_*cscA*_-*cscA*/P_Fru_-UTR_Fru_::P_*tac*_-synUTR_62,626_/P_*gapA*_-UTR_*gapA*_::P_J23100_-synUTR_*gapA*_/pUC-KGG/pACYC_B4	This study
G102_3HP	*E. coli* W/Δ*cscBKAR*::P_J23100_-synUTR_*cscB*_-*cscB* – P_J23106_-synUTR_*cscK*_-*cscK* – P_*tac*_-synUTR_*cscA*_-*cscA*/P_Fru_-UTR_Fru_::P_*tac*_-synUTR_62,626_/P_*gapA*_-UTR_*gapA*_::P_J23102_-synUTR_*gapA*_/pUC-KGG/pACYC_B4	This study
G107_3HP	*E. coli* W/Δ*cscBKAR*::P_J23100_-synUTR_*cscB*_-*cscB* – P_J23106_-synUTR_*cscK*_-*cscK* – P_*tac*_-synUTR_*cscA*_-*cscA*/P_Fru_-UTR_Fru_::P_*tac*_-synUTR_62,626_/P_*gapA*_-UTR_*gapA*_::P_J23107_-synUTR_*gapA*_/pUC-KGG/pACYC_B4	This study
G108_3HP	*E. coli* W/Δ*cscBKAR*::P_J23100_-synUTR_*cscB*_-*cscB* – P_J23106_-synUTR_*cscK*_-*cscK* – P_*tac*_-synUTR_*cscA*_-*cscA*/P_Fru_-UTR_Fru_::P_*tac*_-synUTR_62,626_/P_*gapA*_-UTR_*gapA*_::P_J23108_-synUTR_*gapA*_/pUC-KGG/pACYC_B4	This study
G115_3HP	*E. coli* W/Δ*cscBKAR*::P_J23100_-synUTR_*cscB*_-*cscB* – P_J23106_-synUTR_*cscK*_-*cscK* – P_*tac*_-synUTR_*cscA*_-*cscA*/P_Fru_-UTR_Fru_::P_*tac*_-synUTR_62,626_/P_*gapA*_-UTR_*gapA*_::P_J23115_-synUTR_*gapA*_/pUC-KGG/pACYC_B4	This study
**Plasmids**		
pCDFDuet-1	Expression vector, CloDF13 ori, *Sm*^R^	Novagene
pCDF-CS	CloDF13 ori, *Sm*^R^, P_J23100_-P_*Cm*_-*Cm*^R^-*sacB*-term	This study
pCDF-csc_poly	CloDF13 ori, *Sm*^R^, P_*tac*_-synUTR_*cscA*_-*cscA*-synUTR_*cscK*_-*cscK*-synUTR_*cscB*_-*cscB*	This study
pCDF-csc_mono	CloDF13 ori, *Sm*^R^, P_J23100_-synUTR_*cscB*_-*cscB* – P_J23106_-synUTR_*cscK*_-*cscK* – P_*tac*_-synUTR_*cscA*_-*cscA*	This study
pUC/K	pMB1 ori, *Kan*^R^, P_*tac*_-synUTR_*kgsadh*_-*kgsadh*	(40)
pUC-KGG	pMB1 ori, *Kan*^R^, P_*tac*_-synUTR_*kgsadh*_-*kgsadh* – P_*tac*_-synUTR_*gpd1*_-*gpd1* – P_*tac*_-synUTR_*gpp2*_-*gpp2*	This study
pACYC_B4	p15A ori, *Cm*R, P_*tac*_-synUTR4_*dhaB1*_-*dhaB1*- P_*tac*_-synUTR_*dhaB2*_-*dhaB2*-*dhaB3*-*gdrA*-*gdrB*	(40)

### Construction of the synthetic expression cassettes

Mach1-T1^R^ cells were used for general cloning and cultured in Luria-Bertani (LB) medium containing appropriate antibiotics. All recombinations were done using the DNA fragment from pCDF-CS; selection with chloramphenicol for the 1st recombination (replacement with the *Cm*^R^-*sacB* (CS)-cassette), then counter-selection with sucrose for the final recombination (replacement the CS-cassette in genome with editing template) [17]. Primers used for recombination in this study are listed in Supplementary materials, Table S1.

To construct pCDF-csc_mono, *cscB*_mono, *cscK*_mono, and *cscA*_mono were ligated after cut by NcoI and PstI, after each DNA fragment was amplified by cscB_mono-F/cscB _NcoI-R, cscK _NcoI-F/cscK _PstI-R, and cscA_PstI-F/cscA_mono-R, respectively. Then, each *cscB*-*cscK*-*cscA* cassette and pCDFDuet-1 vector was amplified by cscB_mono-F/cscA_mono-R and pCDF-F/pCDF-R, respectively, then ligated through Gibson Assembly. To construct pCDF-csc_poly, cscA_poly, cscK_poly, and cscB_poly were ligated after cut by SpeI and KpnI, after each DNA fragment was amplified by cscA_PstI-F/cscA_SpeI-R, cscK_SpeI-F/cscK_KpnI-R, and cscB_KpnI-F/cscB_BamHI-R, respectively. Then, each *cscAKB* cassette and pCDF-csc_mono was amplified by cscA_PstI-F/cscB_BamHI-R and pCDF_BamHI-F/pCDF_PstI-R, respectively, then ligated after cut by BamHI and PstI.

To construct pUC-KGG, GPD1 was first cloned to pUC/K. The DNA fragments were ligated after cut with NheI and NdeI, after GPD1 was amplified with GPD1_NdeI-F/GPD1_NheI-R, and pUC/K was amplified with pUC/K_NheI-F/pUC/K_NdeI-R. Then, GPP2 was cloned into the vector after amplified with GPP2_EcoNI-F/GPP2_NheI-R and cut by EcoNI and NheI.

### Media and culture conditions

The cells were cultured in a 250-mL baffled flask containing 25 mL of medium, and incubated at 37 ºC with shaking (250 rpm). The modified minimal medium contained 0.5 g/L MgSO_4_⋅7H_2_O, 2 g/L NH_4_Cl, 2 g/L NaCl, 1 g/L yeast extract, and 100 mM potassium phosphate buffer (pH 7.0); 20 g/L sucrose or 8 g/L of each sucrose, glucose, and fructose for flask-scale cultivation, and 30 g/L of sugarcane molasses with the feed (30 g/L of sugarcane molasses and 4 µM coenzyme B_12_) for fed-batch cultivation were included as the carbon source. To maintain the plasmids, 50 µg/mL of kanamycin, 34 µg/mL of chloramphenicol, or/and 50 µg/mL of spectinomycin was added. To adjust pH, 10 M NaOH solution was used. For 3-HP production, 0.1 mM of IPTG and 4 µM of coenzyme B_12_ were added at OD_600_ 0.7. The culture was conducted with three biological replicates for flask-scale cultivation.

### Metabolite quantification

Concentrations of sucrose, glucose, fructose, 3-HP, glycerol, and acetate were measured at 40 °C using high-performance liquid chromatography (Agilent 1260 Infinity II LC System) equipped with Agilent Hi-Plex H column (8 μm, 6.5 mm × 300 mm, PL1F70-6830) at a flow rate of 0.6 mL/min using 5 mM H_2_SO_4_ as the mobile phase. Signals were monitored at 210 nm of wavelength for UV-Vis and at 35 °C for refractive index detector (RID). Each sample had a 30-min running time. At least five data points of each biochemical were used to draw a standard curve to measure the concentrations.

### Fermentation conditions

Fed-batch fermentation was done in a 5-L bioreactor (BioCNS) containing initial 1.5 L of medium at 37 °C, 250 rpm. pH was controlled with 1 M of NaOH to set at 6.7-7.0. And 30 g/L of sugarcane molasses and 4 µM of coenzyme B_12_ were fed every 24 h [18]. Air was provided at an aeration rate of 0.667 vvm. An electronic zero (0%) on the bioreactor was set first for the DO probe calibration. Prior to inoculation, once the fermentation media was prepared and maintained at 37 °C with agitation at 250 rpm, the DO level was set to 100%. Also, 0.01% of Antifoam 204 (Sigma-Aldrich) was added to the medium prior to the fermentation. The DO level was controlled to 30% of air saturation by automatically increasing the agitation from 200 to 500 rpm first, and then increasing the aeration rate from 0.667 vvm to 3.333 vvm when the agitation was not enough for the control.

## Results and discussion

### Enhancing sucrose consumption rate

The varied consumption pattern of sugars in *E. coli* W, notably delayed sucrose uptake and fructose catabolite repression, stems from the strain’s transport and metabolic characteristics [7, 8]. Sucrose, relying on the non-PTS, requires an additional hydrolysis step, hindering its immediate utilization (Supplementary Fig. S1). Meanwhile, glucose and fructose, facilitated by the PTS, exhibit a more efficient uptake [15]. The sequential sugar consumption reflects the regulatory hierarchy in W, prioritizing glucose followed by fructose. Additionally, pH control in the cultivation environment was required to consume other sugars than glucose.

**Fig. 1:**
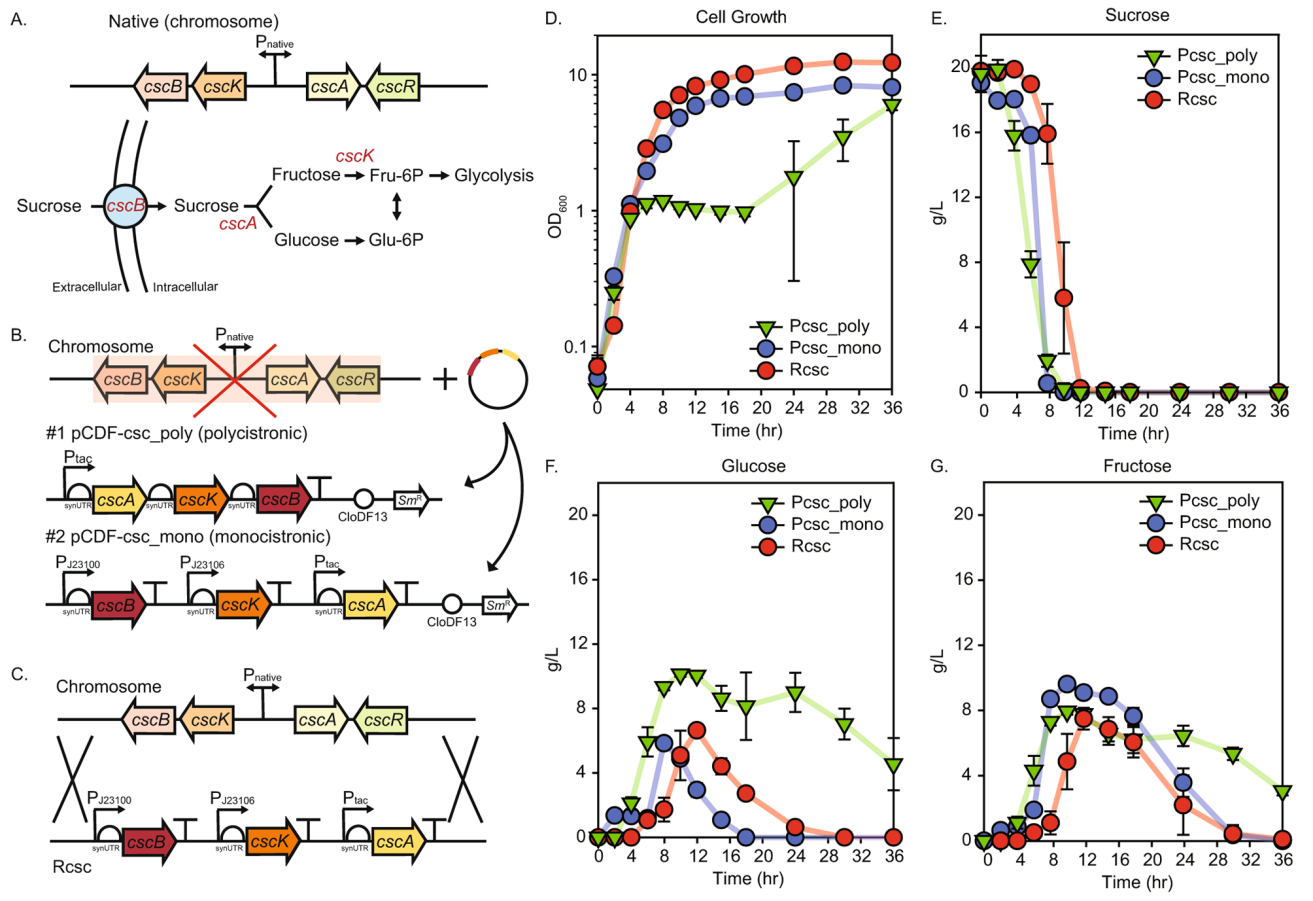
Enhancing the sucrose consumption rate by optimizing the expression level of *csc* operon. (**A**) *csc* operon in native genome, (**B**) plasmid design of the synthetic *csc* operon cassette for both polycistronic and monocistronic expression, with the native *csc* operon-knocked out strain, (**C**) genome-integration of the synthetic *csc* operon; physiology tests of Pcsc_poly (synthetic polycistronic Csc expression through plasmid, green), Pcsc_mono (synthetic monocistronic Csc expression through plasmid, blue), and Rcsc (synthetic monocistronic Csc expression through recombination, red) (**D**) cell growth, (**E**) sucrose consumption, (**F**) glucose consumption, (**G**) fructose consumption graphs

We first engineered the uptake system of the primary component of sugarcane molasses, sucrose, to improve its consumption rate. The main non-PTS sucrose metabolism regulon in W is Csc regulon harboring four genes: *cscB* (permease), *cscK* (fructokinase), *cscA* (invertase), and *cscR* (transcriptional repressor) (Fig. 1A). To augment the sucrose consumption rate, a strategic approach was implemented by initially targeting the transcriptional repressor [7, 19]. While the deletion of *cscR* within the strain was proven effective in enabling complete sucrose consumption, there appeared to be no noticeable differences in the sucrose uptake rate (Supplementary Fig. S2). This observation suggested that the absence of *cscR* might primarily influence the regulatory aspects of the sucrose metabolism pathway, facilitating enhanced utilization without necessarily impacting the rate of sucrose uptake.

To optimize the expression level of *csc* operon, two different expression cassettes were constructed (polycistronic (Pcsc_poly) and monocistronic (Pcsc_mono)) based on a plasmid with a medium copy number (Fig. 1B and Table 1). The polycistronic cassette was designed, featuring a synthetic robust promoter, P_*tac*_, and synthetic 5’ UTRs, with the intent of enhancing the overall operon expression (Supplementary Table S2) [20, 21]. *cscA* and *cscK* were strategically positioned upstream of *cscB*, the membrane protein, mirroring the arrangement in the native operon where they follow a bidirectional promoter. Conversely, the monocistronic cassette involved the individual construction of *cscB*, *cscK*, and *cscA*, each equipped with synthetic 5’ UTRs and constitutive promoters, specifically J23100, J23106, and P_*tac*_, respectively. Based on Registry of Standard Biological Parts [22], the promoter strength in *E. coli* ranks as follows: P_*tac*_, J23100, and J23106, in descending order [23–25]. Additionally, synthetic 5’ UTRs for both polycistronic and monocistronic cassettes exhibited significantly higher translation rates compared to native 5’ UTRs. Overexpression of *cscB* led to an increased presence of permease molecules on the cell membrane, which facilitated the more rapid import of sucrose into the cell. Simultaneously, overexpression of *cscA* enabled the cell to more efficiently hydrolyze the imported sucrose into glucose and fructose, which could then be swiftly utilized in central metabolic pathways like glycolysis. This process helped sucrose entering the cell to break down quickly and to be metabolized, avoiding potential bottlenecks in utilization. Combined, the overexpression of *cscB* and *cscA* optimized both the uptake and conversion of sucrose, resulting in a more efficient and accelerated consumption rate [7]. However, the strength of the promoter for *cscK* was not as strong as for other genes because the overexpression of *cscK*, being a kinase requiring ATP for phosphorylation, exhibited a threshold effect due to limitations in ATP availability. Also, each gene was codon-optimized at the N-terminal for higher expression level, and synthetic strong terminators were used [26] (Supplementary Table S1). These plasmids were subsequently introduced into the *cscBKAR*-deleted strain, thereby creating a targeted genetic context conducive to examining the impact of altered expression patterns on sucrose metabolism. Pcsc_poly exhibited a less favorable performance attributed to potential growth burden, likely led by disrupted metabolic flux (Fig. 1D-G).

The superior performance of Pcsc_mono, characterized by its absence of growth burden and accelerated sucrose consumption rate, proved its selection as the optimal genetic configuration. The synthetically designed monocistronic *csc* cassette was integrated into the *cscBKAR*-deleted genome (Rcsc) to maintain its stable expression and minimize the metabolic burden caused by a heterologous plasmid (Fig. 1C). Despite the deployment of a lower copy number, Rcsc exhibited stable consumption of sucrose and better cellular growth than Pcsc_mono (Fig. 1D-G). Furthermore, the overexpression of invertase expedited the cleavage of sucrose into glucose and fructose, facilitating their rapid transport to the extracellular space (Fig. 1F, G). While this alternation might diverge from a conventional sucrose uptake mechanism, it aligned with the physiological context of sugarcane molasses, which contains a mixture of sucrose, glucose, and fructose. Nevertheless, further engineering is required for the removal of CCR. Enhanced sucrose consumption efficiency, reduced growth burden, and sustained metabolic stability, Rcsc is an advantageous candidate for bioprocess applications requiring optimized sucrose utilization and metabolic control.

### Improving glucose-fructose co-utilization

The removal of the fructose catabolite repression in W was pursued by deleting the transcriptional repressor, *fruR*, also recognized as *cra* in numerous bacteria [27]. This genetic modification successfully abolished CCR on fructose, resulting in a faster fructose consumption rate than glucose (Supplementary Fig. S3). However, a notable consequence of WΔ*fruR* was the accumulation of acetate, indicative of potential disruptions in metabolic flux. The observed accumulation suggested that Cra, catabolite repressor/activator, as a global transcriptional regulator, might exert control over multiple cellular pathways beyond fructose metabolism. This result underscored the need for a balanced approach when manipulating global regulators, as their deletion could inadvertently impact various metabolic pathways, potentially leading to undesirable byproducts [28, 29].

**Fig. 2:**
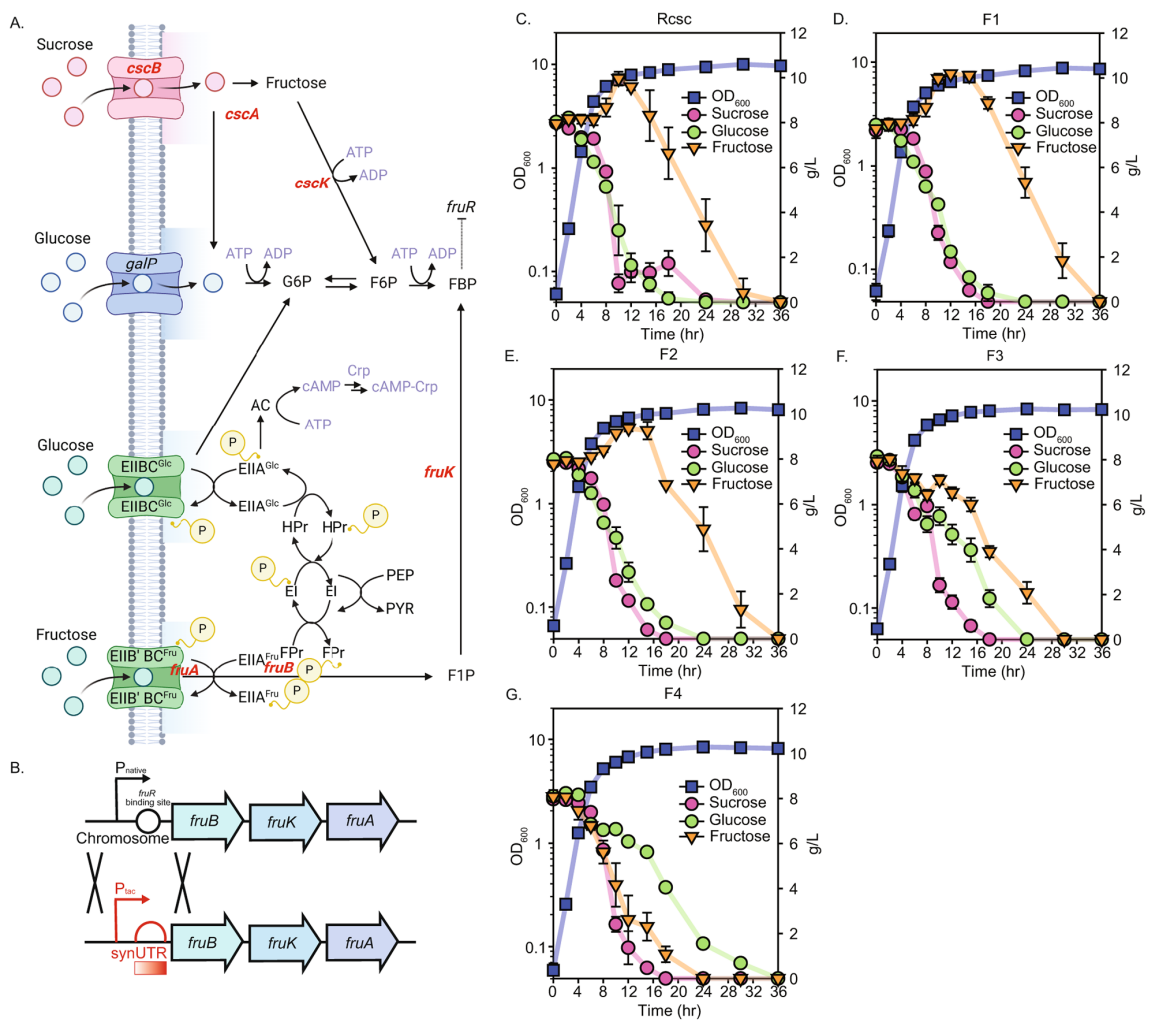
Co-utilization of glucose and fructose by controlling the expression level of fructose PTS regulon with the synthetically designed promoter-5’ UTR cassettes. (**A**) sucrose, glucose, and fructose uptake system in W strain, (**B**) replacement of the native Fru regulon promoter-5’ UTR cassette containing the *fruR* binding site with the synthetic *tac* promoter-5’ UTR cassette, along with 5’ UTR library; physiology tests for cell growth (blue), sucrose consumption (pink), glucose consumption (green), and fructose consumption (orange) of (**C**) Rcsc, (**D**) F1, (**E**) F2, (**F**) F3, (**G**) F4 strains

Instead of direct deletion of *fruR*, a refined strategy was employed to control CCR on fructose by modulating the binding dynamics of FruR with the fructose PTS regulon [30]. The regulon is composed of *fruA*, *fruB*, and *fruK*; FruAB, the fructose PTS permease, also known as the Enzyme II^Fru^ complex, and *fruK*, fructose-1-kinase (Fig. 2A). We replaced the native promoter, operator, and 5’ UTR with a synthetically designed promoter-5’ UTR cassette (Fig. 2B). Furthermore, 5’ UTR library was employed to fine-tune both CCR control and consumption rates, and the strains were labeled based on their predicted translation rates, with F1 representing the lowest and F4 the highest (Table 2) [20, 21]. F1 and F2 demonstrated limited efficacy in alleviating CCR, similar to the control strain presumably due to low translational rates of enzymes (Fig. 2C-E). Conversely, F4 exhibited an accelerated uptake rate of fructose, surpassing that of glucose due to an excessively high translational rate (Fig. 2G). Based on the expression level of Fru regulon, the glucose consumption rate exhibited variability, likely due to competition for phosphoenolpyruvate (PEP) [31]. As the fructose uptake rate increased, the glucose uptake rate correspondingly decreased (Fig. 2D-G). Furthermore, glucose was still consumed even after the hydrolysis of sucrose (Supplementary Fig. S3). These observations suggest the presence of competition for phosphorylation within the cell, particularly involving PEP [32]. The optimal strain, F3, showed effective CCR removal (Fig. 2F). F3 demonstrated near-equivalent co-utilization of glucose and fructose, coupled with the highest sugar consumption rate, underscoring its potential as an industrially relevant variant for enhanced sugar metabolism.

**Table 2:** 5’ UTR sequences for expression of Fru regulon

Strain	5’ UTR sequence (5’-3’)^*^	Predicted expression level (a.u.)
F1	CTTTTTCACTATTTATTCACACAT	3,231
F2	TCAAATAAAATCTAAGAAAACAAGAATC	12,136
F3	CAGACTATCTATCTATAAGGTTTTTTT	62,626
F4	AGCTACAAAGCCAACAACAATAAGGAGTTTTTTA	1,386,568

^*^Underlined letters indicate the ribosome binding site

With the alleviation of CCR, there were more carbon sources available for metabolism, leading to the accumulation of acetate (Supplementary Fig. S4). Due to the increased flow rate, acetate could not be eliminated completely, but a reduction by approximately 16% in the F3 strain was observed at 30 h., just before acetate becoming the primary carbon source. FruR is modulated by the concentration of fructose-1,6-biphosphate (FBP) produced by *fruK* [33]. However, some research suggested that there was no direct relationship between FruR and FBP, indicating that FruR is only affected by fructose-1-phosphate (F1P) [34]. Despite these controversies regarding whether FruR is regulated by FBP, it is clear that FBP is directly regulated by *fruK* and F1P which is directely influenced by FruR. Constructing a synthetic cassette of the fructose PTS regulon independent of *fruR* expression enhanced fructose metabolism but could potentially disrupt overall cellular metabolism [35]. FruR regulates approximately 79 regulons, some of which are other transcription factors [36, 37]. Continuous production of FBP can deactivate FruR, potentially leading to the overexpression of genes unrelated to fructose metabolism and a consequent waste of cellular resources. Further improvements might be achieved by inducing mutations in *fruR* or through adaptive laboratory evolution (ALE) to enhance overall metabolic efficiency [38]. The integration of simultaneous sugar uptake strategies thus represents a promising avenue for biotechnological applications.

### Optimizing 3-HP production

**Fig. 3:**
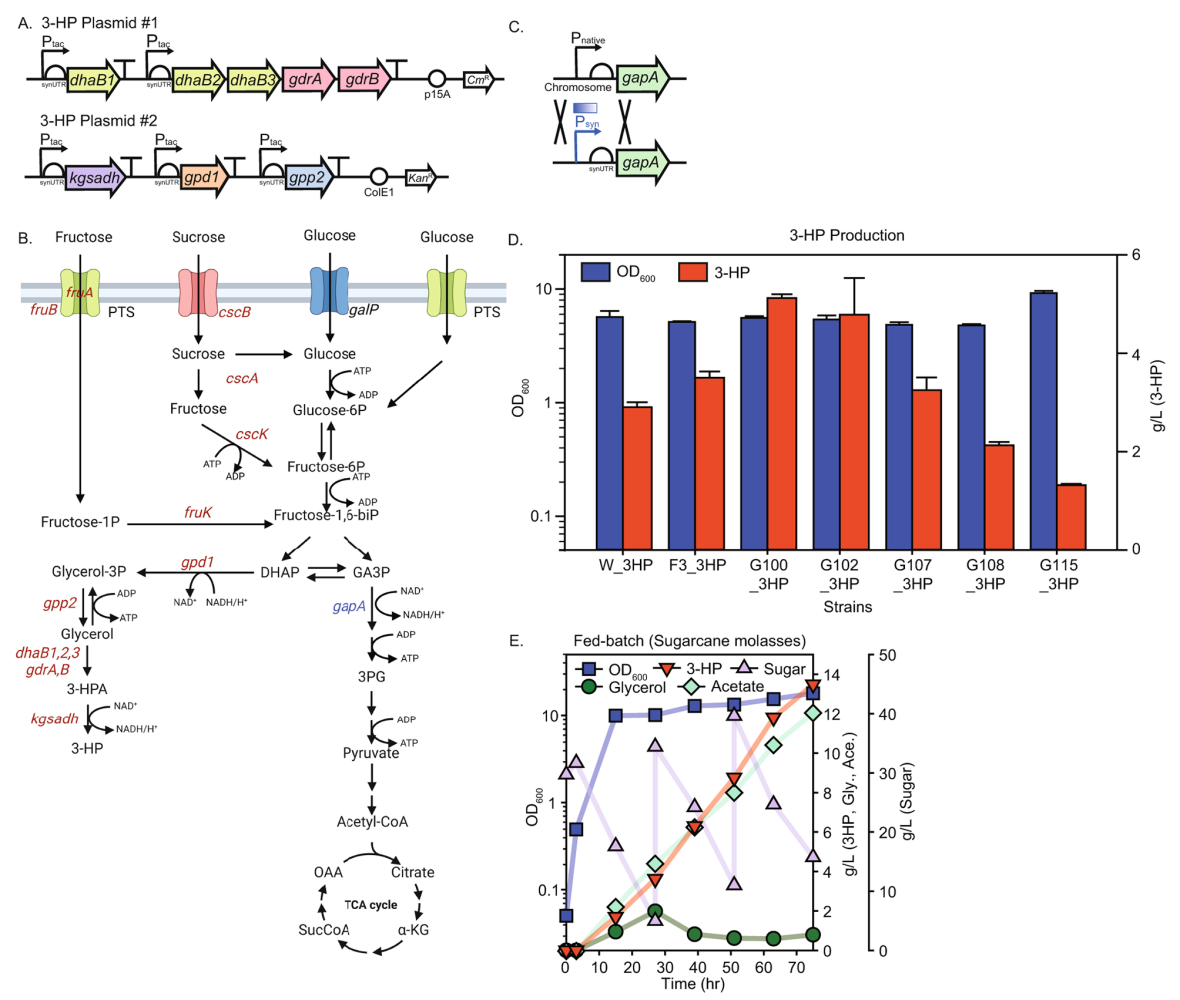
3-HP production by optimizing the expression level of *gapA*. (**A**) synthetic design of the 3-HP pathway plasmids, (**B**) 3-HP pathway from sucrose, glucose, and fructose along with the enzymes (red, over-expression; blue, repression) that were engineered in the 3-HP producing strain, (**C**) replacement of the native *gapA* promoter-5’ UTR cassette with the synthetic promoter-UTR cassette, along with promoter library, (**D**) 3-HP production (red) bar graph of each strain with cell growth (blue), (**E**) fed-batch fermentation of the final strain, G100_3HP, using sugarcane molasses as a substrate; cell growth (blue), 3-HP (red), sugar, combination of sucrose, glucose, and fructose (purple), glycerol (green), and acetate (mint)

To validate the industrial applicability of strains exhibiting increased sucrose consumption rate while simultaneously being able to consume multiple carbon sources, 3-HP was produced using raw material, sugarcane molasses. Multiple heterologous genes were introduced for the construction of the 3-HP pathway in the engineered strain. Harnessing the glycerol pathway as the fundamental route for 3-HP biosynthesis from sugarcane molasses, renowned for its efficiency and productivity, the selection of this pathway was based on the identification of the required enzymes facilitating the conversion from sucrose to glycerol [18]. To convert the carbon sources from sucrose to glycerol, two genes coding for glycerol-3-phosphate dehydrogenase (*gpd1*) and glycerol-3-phosphatase (*gpp2*) were expressed [39]. Also, from glycerol to 3-HP, two enzymatic reactions were required, dehydration of glycerol to 3-hydroxypropionaldehyde (3-HPA) and oxidation of 3-HPA, which were catalyzed by glycerol dehydratase (GDHt) and aldehyde dehydrogenase (ALDH), respectively. Synthetically designed 3-HP pathway genes were used in this study to rebalance the pathway for preventing the accumulation of 3-HPA which was toxic, from the previous study [40]. Two plasmids, each designed to accommodate and drive the overexpression of the pathway genes – namely *gpd1*, *gpp2*, *dhaB1*,*2*,*3*, *gdrA*,* B*, and *kgsadh* – were used (Fig. 3A and B).

Previous studies reported that downregulating or replacing native GAPDH to enhance NAD(P)H availability and mitigate futile cycles between endogenous and heterologous enzymes [41, 42]. To assess the potential improvement in 3-HP production, the expression level of *gapA* was rebalanced with alterations in the upstream carbon flux. This targeted repression aimed to reduce glycolytic pathway fluxes, concurrently enhancing 3-HP production through the glycerol pathway [43, 44]. The expression level of the glycolytic enzyme, *gapA* was diversified via a promoter library: J23100, J23102, J23107, J23108, J23115 (Fig. 3C) [45]. This regulatory intervention yielded a 1.76-fold increase in the 3-HP titer in G100_3HP strain compared to the control strain (Fig. 3D). Excessive repression of *gapA* (G115_3HP) yielded suboptimal physiological outcome, highlighting the importance of identifying the optimal flux. Compared to the previous studies utilizing a glycerol pathway and sugar(s) as substrates in *E. coli* for 3-HP production, this study demonstrated a significant elevated titer, yield, and productivity in batch culture : 5.123 g/L, 0.337 g/g, and 0.142 g/L-h [46, 47].

Fed-batch cultivation of G100_3HP strain with a substrate of sugarcane molasses demonstrated the 3-HP titer of 13.487 g/L and the productivity of 0.180 g/L-h (Fig. 3E; Table 3). In the results of the fed-batch culture, accumulation of acetate was observed, likely due to an overabundance of glycolytic substrates, which demanded a greater presence of respiratory chain proteins to produce ATP through respiration [48]. This could result in a higher proteomic cost for ATP production via respiration than by fermentation. Also, the acetate accumulation might be caused by variations in the composition and amount of multiple carbon sources present in sugarcane molasses [49, 50]. To mitigate acetate accumulation and enhance 3-HP production, redirecting the flux of carbon sources toward cell growth and 3-HP production, rather than byproduct formation, could improve yield. Potential strategies include the overexpression of acetyl-CoA synthetase (*acs*) to convert acetate to acetyl-CoA, or the deletion of pyruvate oxidase (*poxB*) to prevent the conversion of pyruvate to acetate [51].

**Table 3:** Comparison of titer, yield, and productivity of each 3-HP producing strain

Strain	Titer (g/L)	Yield (g/g)	Productivity (g/L-h)	Substrate	Cultivation
W_3HP	2.904	0.172	0.081	Sucrose (8 g/L) + glucose (8 g/L) + fructose (8 g/L)	Shake-flask/batch
F3_3HP	3.504	0.248	0.097		
G100_3HP	5.123	0.337	0.142		
G102_3HP	4.634	0.337	0.129		
G107_3HP	3.251	0.144	0.090		
G108_3HP	2.128	0.105	0.059		
G115_3HP	1.319	0.096	0.037		
G100_3HP	13.487	0.182	0.180	Sugarcane molasses (30 g/L, with 30 g/L feed)	Bioreactor/fed-batch

Despite reducing the expression level of *gapA*, the presence of available glucose (additional glucose from sucrose breakdown) might induce overflow metabolism to manage the excess resources. Optimization of metabolic flux through a systematic approach could further enhance biochemical production [52]. This outcome not only validated the effectiveness of the engineered regulatory control over *gapA*, but also highlighted the potential scalability and industrial viability of G100_3HP strain for advanced 3-HP biosynthesis in a fed-batch cultivation setting. The results showed a step forward in optimizing the production of 3-HP, positioning the engineered strain as a promising candidate for bioconversion processes on a larger scale.

To achieve higher 3-HP titers, genetic engineering efforts had been undertaken, including pathway optimization. This involved the design and implementation of synthetic pathways, to enhance the expression of key pathway genes. Specifically, synthetic promoters and 5’ UTRs had been utilized to drive overexpression of these genes, thereby increasing the flux through the 3-HP biosynthetic pathway. Additionally, flux control mechanisms had been incorporated to redirect metabolic flux more effectively towards 3-HP production. By strategically engineering these pathways and optimizing the associated regulatory element (*gapA*), the overall efficiency and yield of 3-HP production had been improved. However, some fermentation optimizations can be potential further work to improve the titer. To enhance 3-HP production, various parameters could be optimized, including substrate concentration control, two-stage aeration strategies, two-stage pH control, and medium optimization, among others [42, 53–55].

## Conclusions

Despite certain limitations in sucrose metabolism in *E. coli*, the redesigned *E. coli* W strain, proficient in utilizing sugarcane molasses, emerges as a valuable asset for industrial fermentation. The integration of synthetic *csc* operon, enhancing sucrose consumption, and the mitigation of CCR for improved co-utilization of glucose and fructose highlight its significance. Additionally, *gapA* repression contributes to carbon flux redistribution, further amplifying its potential for converting molasses into 3-HP efficiently. This optimized strain not only addresses current limitations in sucrose and fructose metabolism but also paves the way for future applications in sustainable and cost-effective industrial biotechnology.

## Electronic supplementary material

Below is the link to the electronic supplementary material.

Supplementary Material 1

Supplementary Material 2

Supplementary Material 3

Supplementary Material 4

## Data Availability

No datasets were generated or analysed during the current study.
