# Correction: Synthetic redesign of *Escherichia coli* W for faster metabolism of sugarcane molasses

**DOI:** 10.1186/s12934-024-02555-2

**Published:** 2024-10-14

**Authors:** Gi Yeon Kim, Jina Yang, Yong Hee Han, Sang Woo Seo

**Affiliations:** 1 Interdisciplinary Program in Bioengineering, Seoul National University, 1 Gwanak-ro, Gwanak-gu, Seoul, 08826 South Korea; 2 Department of Chemical Engineering, Jeju National University, 102, Jejudaehak-ro, Jeju-si, Jeju-do, 63243 Korea; 3 School of Biological Sciences and Biotechnology, Graduate School, and School of Biological Sciences and Technology, Chonnam National University, Yongbong-ro 77, Gwangju, 61186 South Korea; 4 School of Chemical and Biological Engineering, Seoul National University, 1 Gwanak-ro, Gwanak-gu, Seoul, 08826 South Korea; 5 Institute of Chemical Processes, and Bio-MAX Institute, and Institute of Bio Engineering, Seoul National University, 1 Gwanak-ro, Gwanak-gu, Seoul, 08826 South Korea

**Correction: Microbial Cell Factories (2024) 23: 242**

10.1186/s12934-024-02520-z.

In this article, Gi Yeon Kim and Jina Yang should have been denoted as equally contributing authors.

The original article has been corrected.

