# Supplementary material for: Synthetic redesign of Escherichia coli W for faster metabolism of sugarcane molasses
Source: Microb Cell Fact. 2024 Sep 9;23:242. doi: 10.1186/s12934-024-02520-z (PMC11382391; doi:10.1186/s12934-024-02520-z)
Supplement: Supplementary file 2 — Supplementary Material 2 [file 12934_2024_2520_MOESM2_ESM.pdf]

Additional file 1

Supplementary Figure S1. CCR test on wild type W with the pH control using sucrose, glucose, and fructose as substrates. (A) CCR confirmation test of sucrose, glucose, and fructose on wild type W with pH control, (B) CCR confirmation test of sucrose, glucose, and fructose on wild type W without pH control.

Supplementary Figure S2. Physiology test between wild type W and WΔ*cscR* strains using sucrose as a substrate. (A) cell growth, (B) sucrose consumption graphs.

Supplementary Figure S3. Physiology tests between wild type W and WΔ*fruR* strains using sucrose, glucose, and fructose as substrates. (A) cell growth, (B) acetate accumulation, (C) sucrose consumption, (D) glucose consumption, (E) fructose consumption graphs.

Supplementary Figure S4. Acetate accumulation comparison between WΔ*fruR*, F1, F2, F3, and F4 strains using sucrose, glucose, and fructose as substrates.

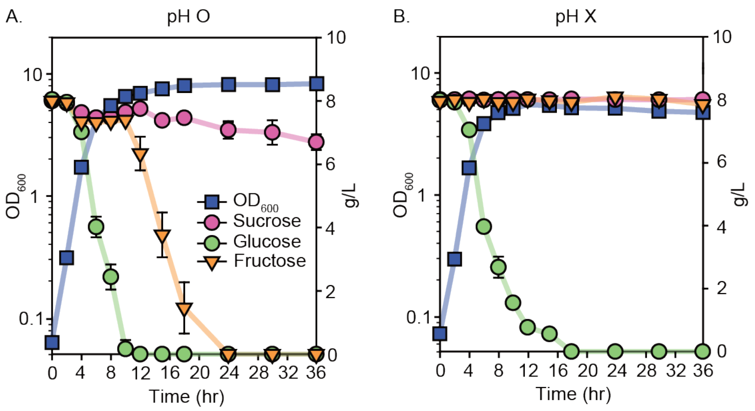

Supplementary Figure S1. CCR test on wild type W with the pH control using sucrose, glucose, and fructose as substrates. (A) CCR confirmation test of sucrose, glucose, and fructose on wild type W with pH control, (B) CCR confirmation test of sucrose, glucose, and fructose on wild type W without pH control.

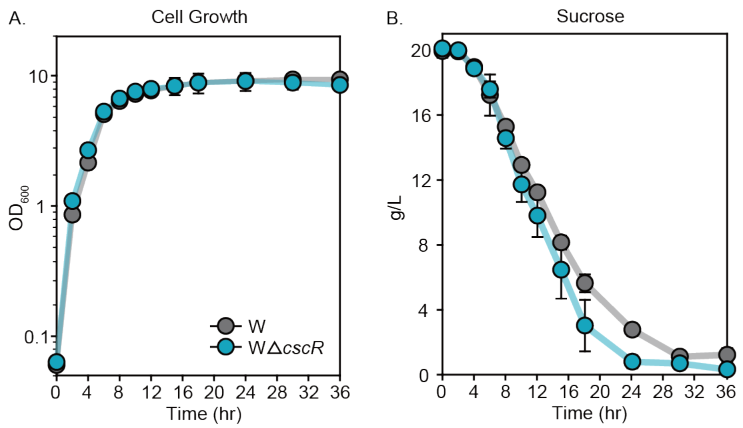

Supplementary Figure S2. Physiology test between wild type W and WΔ*cscR* strains using sucrose as a substrate. (A) cell growth, (B) sucrose consumption graphs.

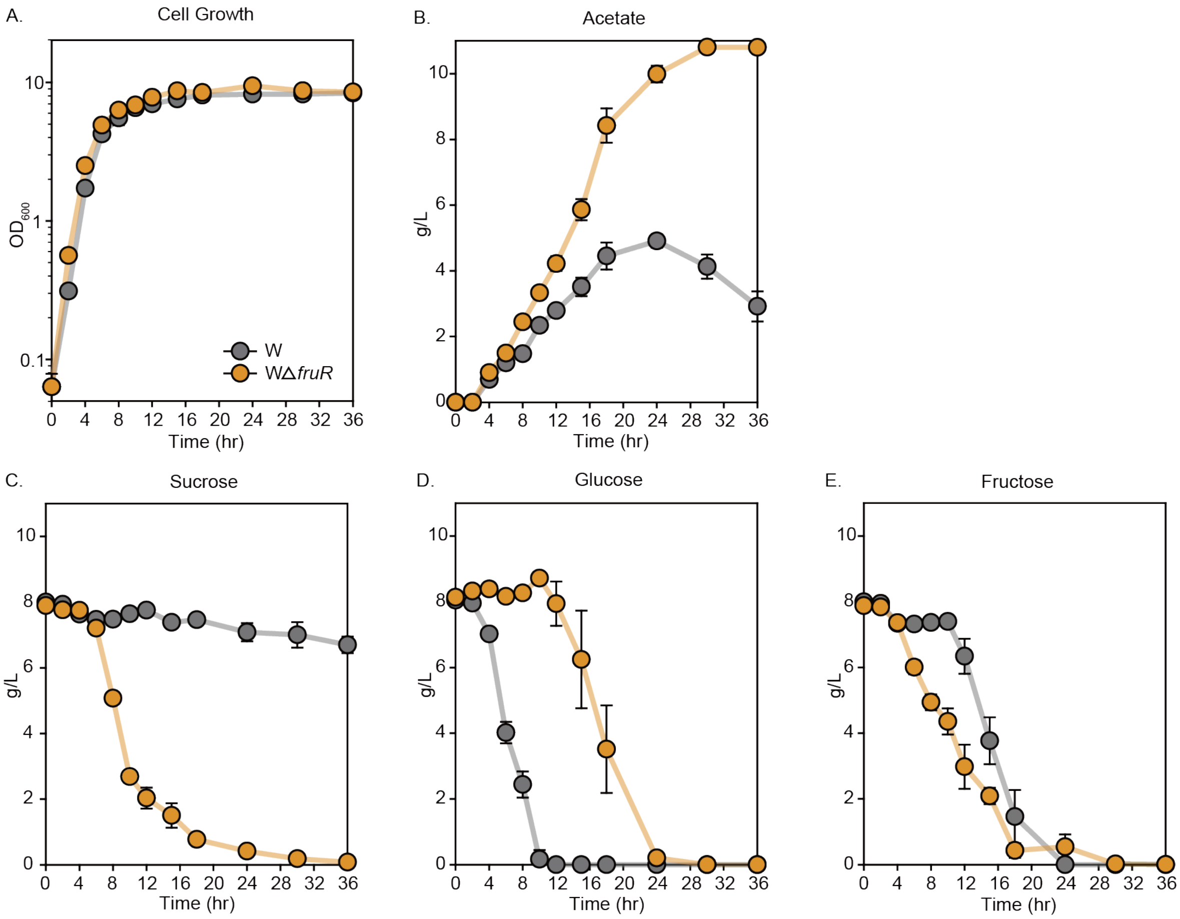

Supplementary Figure S3. Physiology tests between wild type W and WΔ*fruR* strains using sucrose, glucose, and fructose as substrates. (A) cell growth, (B) acetate accumulation, (C) sucrose consumption, (D) glucose consumption, (E) fructose consumption graphs.

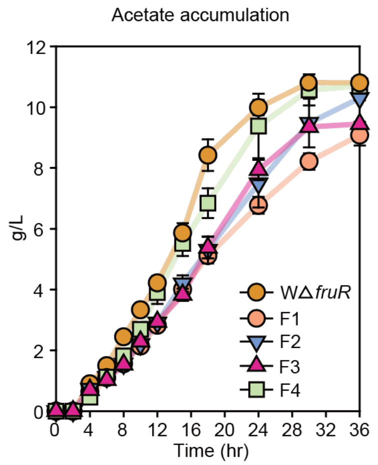

Supplementary Figure S4. Acetate accumulation comparison between WΔ*fruR*, F1, F2, F3, and F4 strains using sucrose, glucose, and fructose as substrates.
